# Red Blood Cell Size Is Inversely Associated with Leukocyte Telomere Length in a Large Multi-Ethnic Population

**DOI:** 10.1371/journal.pone.0051046

**Published:** 2012-12-04

**Authors:** Julia Kozlitina, Christine Kim Garcia

**Affiliations:** 1 Eugene McDermott Center for Human Growth and Development, University of Texas Southwestern Medical Center, Dallas, Texas, United States of America; 2 Department of Internal Medicine Division of Pulmonary and Critical Care Medicine, University of Texas Southwestern Medical Center, Dallas, Texas, United States of America; University of North Carolina, United States of America

## Abstract

Although mutations in the genes encoding either the protein or RNA component of telomerase have been found in patients with various blood disorders, the impact of telomere length on hematopoiesis is less well understood for subjects from the general population. Here we have measured telomere lengths of genomic DNA isolated from circulating leukocytes of 3157 subjects, ranging from 18 to 85 years of age, enrolled in a large multiethnic population based study, the Dallas Heart Study 2. Shorter telomere lengths are marginally associated with lower red blood cell counts in this cohort, but are significantly associated with larger mean red blood cell size (as measured by the MCV), increased red blood cell distribution width (RDW), higher hemoglobin levels and lower platelet counts, even after correction for age, gender and ethnicity (p-values of <0.0001, <0.0001, 0.0009 and 0.0016, respectively). In a multiple regression model we find that telomere length is a significant covariate of MCV (p = 7.6×10^−8^), independent of age, ethnicity, BMI, current smoking, alcohol consumption, iron or homocysteine levels. The effect of telomere length on MCV variation is comparable to the effect of smoking or alcohol consumption and is more significant in older individuals (p = 9.2×10^−7^ for >50 years vs. p = 0.0006 for <50 years of age). To our knowledge, this is the first report of an association between telomere length and red cell size in a large urban US population and suggests a biologic mechanism for macrocytosis of aging.

## Introduction

Telomeres are specialized structures that stabilize the ends of chromosomes [1]. The multimeric ribonucleoprotein enzyme telomerase catalyzes the addition of tandem sequence repeats to the telomeres so as to maintain their length after multiple rounds of DNA replication [2]. In humans the protein component of telomerase is expressed only in cells with the capacity to replicate [3] and its activity is globally restricted so that there is progressive shortening of telomere length with age [4]. Since telomere shortening can lead to senescence and then “crisis” or arrest of cultured cell growth [5], telomere length has been proposed to be a “mitotic clock,” or a surrogate marker of cellular age [6].

The relevance of telomere shortening to human physiological age is suggested by the phenotypes of patients with rare mutations in the genes encoding the telomerase complex. Patients with dyskeratosis congenita (DC) develop mucocutaneous features of the disease during infancy, followed by bone marrow failure and premature death [7]. While DC is the most severe clinical manifestation of telomerase dysfunction, rare germline mutations in the telomerase genes (*TERT* and *TERC*) have also been found in patients with isolated aplastic anemia [8], myelodysplastic syndrome [9], acute myeloid leukemia [10], pulmonary fibrosis [11], [12] and liver cirrhosis [13], [14]. In kindreds with familial pulmonary fibrosis and germline *TERT* mutations, mutation carriers have a reduced life expectancy with a mean age of death of 58 and 67 for males and females, respectively [15].

Telomere lengths shorten over a wide range of ages in human populations. These lengths reflect the endogenous telomerase activity of the bone marrow progenitor cells as well as effects from exogenous oxidative or inflammatory stressors which can accelerate telomere shortening. Telomere lengths are found to be longer in centenarians and their offspring than controls and have been associated with achievement of higher levels of education and less cognitive decline with age [16]. Conversely, several investigations have determined that short telomere lengths are associated with decreased survival of patients with heart disease, cancer and infections [17], [18], [19], [20], [21].

Previously, we found that subjects with an inherited predisposition to develop pulmonary fibrosis due to germline *TERT* mutations have lower red blood cell (RBC) counts, larger RBC size, as measured by the mean red blood cell volume (MCV), and lower platelet counts than family member controls [22]. Of all the quantitative lung and blood phenotypes that were measured in that study, MCV was the one that was most closely correlated with leukocyte telomere lengths [22]. Given the established association between bone marrow failure syndromes and telomerase mutations in rare patients, we hypothesized that telomere lengths may be associated with certain hematologic phenotypes in the general population. To test this hypothesis, we have explored the associations between telomere length and complete blood count measurements in the large multiethnic population of the Dallas Heart Study 2.

## Results

### Assessment of Telomere Lengths in the DHS2

**Figure 1:**
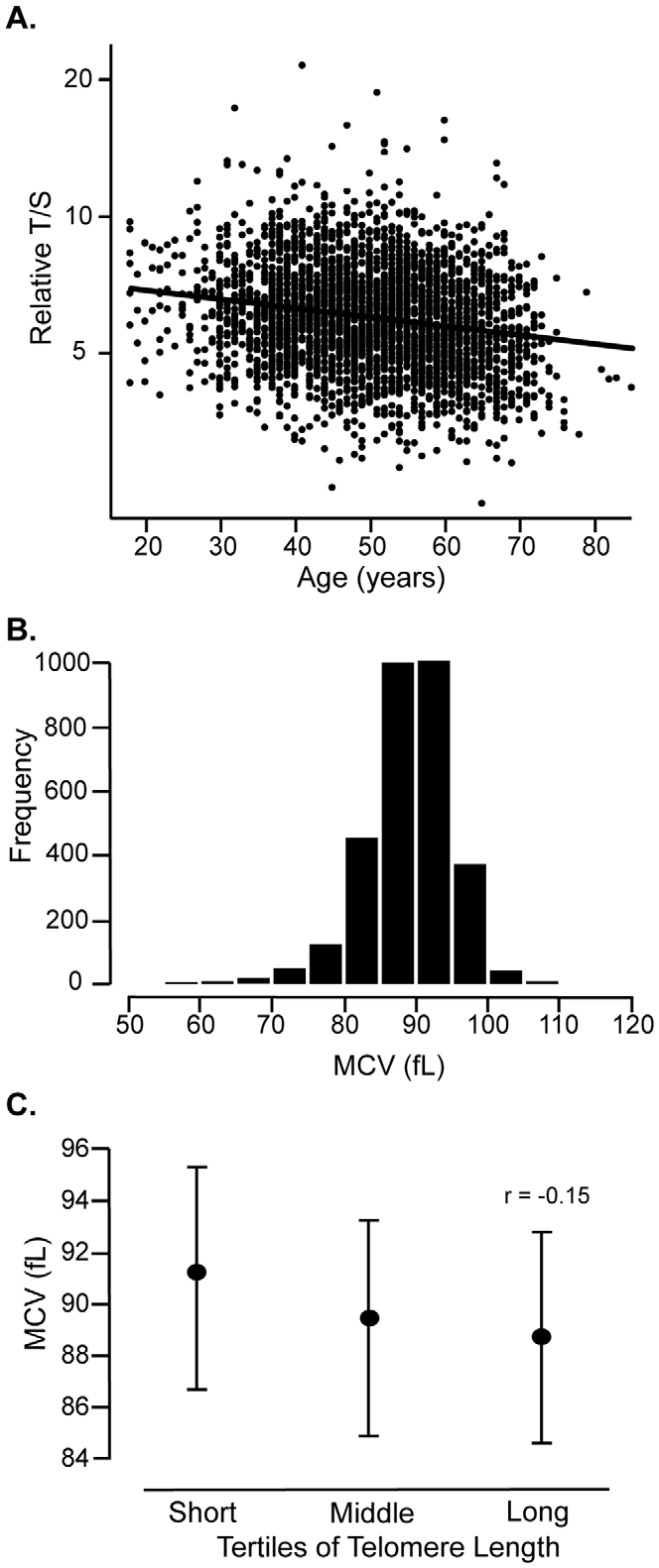
Distribution of Telomere Length and RBC Mean Corpuscular Volume of DHS2 Subjects. (**A**) Each dot represents a subject from the Dallas Heart Study 2 (DHS2). Telomere length is measured from genomic DNA isolated from blood leukocytes using a quantitative PCR assay, expressed as the Relative T/S ratio. The ratio of the copy number of telomere DNA (T) to a single-copy gene (S) for each subject is expressed relative to a cultured cell line (MCF7) which has very short telomere lengths (see Methods for additional details). (**B**) Distribution of red blood cell Mean corpuscular volume (MCV) in the DHS2 population. (**C**) The mean MCV for each of the telomere length tertiles is indicated by the point and the interquartile (1st through 3rd) distribution is indicated by the bars.

Telomere length was measured using a quantitative PCR (qPCR) assay as the ratio of telomere-specific amplification to a single gene as described in the **Materials and Methods**. We find that telomere length decreases with age for DHS2 subjects (**Figure 1A**). The median telomere length is estimated to be 6.27 kb for the entire population. Subjects in this cohort range from 18 to 85 years of age with a median age of 50.0 years. The observed mean coefficient of variance for the qPCR measurement of 3302 samples is 4.09%. To achieve constant variance across the range of telomere lengths and to correct the slight skewness of the qPCR measurements, we have used a logarithm transformation of these values in statistical analyses.

Subjects in the lowest tertile of telomere length are significantly older than those in the middle and upper tertiles (**Table 1**, p<0.0001), therefore all subsequent comparisons are corrected for age. The proportion of males is significantly higher among participants with shorter telomere lengths (p<0.0001). Hispanic, but not African American or European American, ethnicity is associated with telomere length; there are more Hispanics within the longest telomere length tertile after adjustment for gender and age (p = 0.030). There is no difference in total white blood cell counts across tertiles, although the percentage of monocytes is lowest in the group with the longest telomere lengths (**Table S1**). Shorter telomere lengths are associated with lower platelet counts (p = 0.0016) after correction for age, gender and ethnicity. Although shorter telomere length is only marginally associated with decreased RBC counts (p = 0.056), it is strongly associated with larger mean RBC size (as measured by the MCV), greater RBC distribution width (RDW) and higher hemoglobin levels (p-values of <0.0001, <0.0001 and 0.0009, respectively). The distribution of the MCV values as well as the median and the interquartile ranges of MCV for the different telomere length groups are shown in **Figures 1B and 1C**, respectively. There is no association between telomere length and iron levels or between telomere length and homocysteine levels, after adjustment for age, gender and ethnicity. Other clinical characteristics of the DHS2 cohort stratified by telomere length are listed in **Table 1** and **Table S1**.

**Table 1:** Demographic and Clinical Characteristics of the DHS2 Stratified by Leukocyte Telomere Length Tertile.

	First(Shortest)n = 1053	Second(Middle)n = 1052	Third(Longest)n = 1052	*P*-value	*P*-value adjusted for covariates*
Telomere length (kb)	4.14–6.02	6.02–6.53	6.53–9.20		
Age (years)	52.7±11.2	49.2±10.9	48.3±10.8	<0.0001	–
Male (%)	44.3	40.8	36.1	0.0001	<0.0001
African American (%)	52.9	50.3	52.0	0.6792	0.2961
European American (%)	35.4	34.4	31.4	0.0492	0.6530
Hispanic (%)	11.7	15.3	16.6	0.0013	0.0300
WBC count (×10^9^/L)	6.61±2.11	6.72±1.99	6.56±2.14	0.2038	0.6349
RBC count (×10^9^/L)	4.56±0.50	4.60±0.49	4.56±0.47	0.8087	0.0560
Hemoglobin (g/dL)	13.8±1.7	13.7±1.7	13.5±1.6	<0.0001	0.0009
MCV (fL)	90.1±6.7	88.6±6.4	88.1±6.4	<0.0001	<0.0001
RDW (%)	14.1±1.5	14.1±1.7	13.9±1.5	<0.0001	<0.0001
Platelet count (×10^9^/L)	245±69	249±67	259±67	<0.0001	0.0016
Iron (µg/dL)	88.7±35.3	88.3±40.0	86.8±36.1	0.2082	0.6722
Homocysteine	8.94±3.38	8.83±3.48	8.81±4.88	0.0054	0.8474
BMI (kg/m^2^)	31.0±7.2	31.3±7.4	31.5±7.7	0.1952	0.2195
Smoking (%)					
Never	50.0	55.7	57.3	0.0006	0.1020
Former	23.5	23.5	21.2	0.2358	0.2150
Current	26.5	20.7	21.5	0.0092	0.0027
Smoking (pack-years)^ †^	20.7±18.7	18.1±17.6	17.1±15.8	0.0190	0.2665
Alcohol Drinkers (%)					
Never	8.5	8.0	9.8	0.2731	0.4650
Former	21.9	21.5	20.3	0.3957	0.6795
Current	69.6	70.4	69.9	0.7190	0.4162
Alcohol intake (g/day)	0.30 (0–5.6)	0.31 (0–5.6)	0.21 (0–4.2)	0.9387	0.5260

Quantitative variables are shown as mean ± SD or median (1^st^ – 3^rd^ quartile). *P*-values are based on the Jonckheere-Terpstra test for trend or linear regression for continuous variables, and chi-square tests for trend or logistic regression for qualitative variables. ^†^Includes current smokers only. Pack-years are calculated as (packs smoked per day) × (number of years as a smoker).

* Gender is adjusted for age only. Analyses of ethnicities are adjusted for age and gender. All other analyses are adjusted for age, gender and ethnicity.

There is a statistically significant higher percentage of current smokers in the lowest telomere length tertile (p = 0.0027), but no difference in the percentages of former or never smokers across tertiles (**Table 1**). A cumulative effect of smoking (as measured by the pack-years) on telomere length was not significant after correction for age, gender and ethnicity (p = 0.26). We find no association in this population between telomere length and alcohol consumption.

We assessed the effect of different covariates on telomere length in a multiple regression model (**Table 2**). We find that age is the most significant covariant of telomere lengths (p = 1.1×10^−24^), with an estimated attrition rate of 10 basepairs per year. Telomere lengths are about 83 base pairs shorter in males than in females, equivalent to about an 8-year difference in age (p = 9.1×10^−5^). We find that African American ethnicity is not associated with telomere length. Hispanic ethnicity is associated with longer telomere length, even after adjusting for gender and age (p = 0.0069, Hispanic vs. African American; p = 0.0339, Hispanic vs. European American). Current smokers have shorter telomere lengths (approximately 93 base pair difference) than those who have never smoked. Subjects who had smoked in the past had no difference in telomere length from those who had never smoked.

**Table 2:** Clinical Variables Associated with Telomere Length.

	Model adjusted for age, gender, and ethnicity(n = 3157, R^2^ = 0.044)		Full model(n = 3047, R^2^ = 0.048)	
	Beta (SE)	*P*-value	Beta (SE)	*P*-value
Age (per year)	−0.0045 (0.0004)	2.7×10^−27^	−0.0044 (0.0004)	1.1×10^−24^
Gender				
Female	Reference		Reference	
Male	−0.0386 (0.0092)	2.8×10^−5^	−0.0365 (0.0093)	9.1×10^−5^
Ethnicity				
European Am. vs. African Am.	0.0042 (0.0101)	0.6791	0.0091 (0.0106)	0.3945
Hispanic vs. African Am.	0.0274 (0.01334	0.0416	0.0400 (0.0148)	0.0069
Hispanic vs. European Am.	0.0232 (0.0143)	0.1048	0.0310 (0.0146)	0.0339
Smoking				
Never			Reference	–
Past smoking			−0.0064 (0.0116)	0.5844
Current smoking			−0.0406 (0.0114)	0.0004
*TERC* SNP rs2293607			−0.0249 (0.0088)	0.0048

Relative T/S ratios were logarithm transformed prior to analysis. The effect sizes (betas) are reported as mean differences in the logarithm of T/S ratio associated with a one-unit increase in a quantitative predictor, or with a particular category of a qualitative predictor, compared to the reference group. Corresponding changes in T/S ratio are multiplicative, i.e., T/S ratios differ by a factor *e* ^beta^. Interactions between age and gender and between age and ethnicity were not statistically significant and were not included in the final model.

Common variants within the 3' region flanking the gene encoding the telomerase RNA component (*TERC*) have been reported to be associated with telomere length in large populations [23], [24]. The frequencies of the two examined SNPs differ markedly among ethnic groups. Within each ethnic group, the genotype distributions are in Hardy-Weinberg equilibrium (**Table S2 and S3**) and neither SNP shows a consistent association with telomere length among all three ethnic groups (**Figure S1**). We have included only one SNP in the multiple linear regresssion analysis since the pair is in strong linkage disequilibrium (D' = 0.962 and R^2^ = 0.052 in African Americans, D' = 0.997 and R^2^ = 0.870 in European Americans, and D' = 0.994 and R^2^ = 0.616 in Hispanics). The *TERC* SNP rs2293607 is associated with a shortening of about 57 base pairs in telomere lengths in carriers compared to wild-type homozygotes (p = 0.0048). All together, the covariates listed in the full model explain only a fraction of the inter-individual variability in telomere length (R^2^ = 4.8%). We find that there is no evidence of a difference in the rate of age-related telomere length attrition between ethnicities and genders, by including the corresponding interaction terms to the regression model.

### Assessment of Hematologic Parameters in the DHS2

Overall, 13.4% of the DHS2 population has anemia, as defined by a hemoglobin level less than 12 g/dL (**Table 3**). A greater proportion of women than men has microcytic anemia; among women, African-Americans have a higher prevalence of microcytic anemia (10.3%) than either European-Americans (2.4%) or Hispanics (6.3%). We observe a low overall frequency of macrocytosis (MCV >100 fL; 2.1%) in the DHS2 population, which is found more frequently in European-Americans. There is no difference in the frequency of macrocytic anemia in subjects of either gender or of different ethnicities (overall frequency of 0.2%).

**Table 3:** Blood Count Characteristics of the DHS2 participants.

	AfricanAmerican(n = 1633)	EuropeanAmerican(n = 1065)	Hispanic(n = 459)	*P* race			*P* gender
				African vs.European American	African Americanvs. Hispanic	European Americanvs. Hispanic	
Anemia (Hemoglobin <12 g/dL), %				<0.0001	<0.0001	0.0025	<0.0001
Men	4.5	0.8	0.5				
Women	30.0	7.1	14.1				
Microcytosis (MCV <80 fL), %				<0.0001	0.0002	0.0039	<0.0001
Men	6.2	0.6	1.1				
Women	14.3	3.3	7.8				
Microcytic anemia				<0.0001	0.0545	0.0036	<0.0001
Men	0.5	0.0	0.5				
Women	10.3	2.4	6.3				
Macrocytosis (MCV >100 fL), %				0.0009	0.7974	0.0242	0.9495
Men	1.5	3.3	1.6				
Women	1.4	3.7	1.1				
Macrocytic anemia				0.4525	0.9906	0.9910	0.4277
Men	0.2	0.2	0.0				
Women	0.5	0.2	0.0				

* *P*-values are based on logistic regression with adjustment for gender and ethnicity.

**Figure 2:**
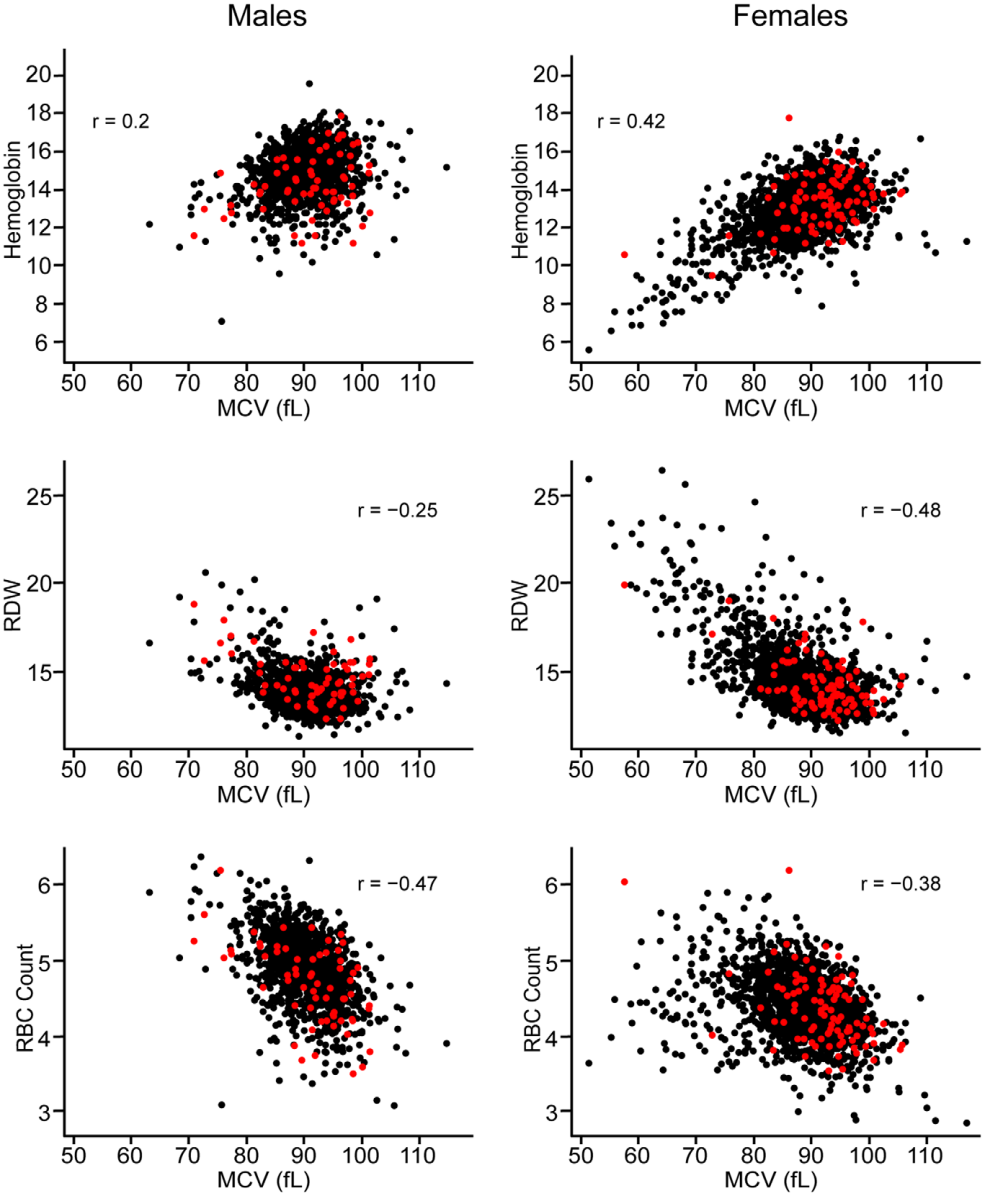
Graphical representation of the relationship between hemoglobin, RDW, RBC count and MCV for the DHS2. Each dot represents a subject from the Dallas Heart Study 2 (DHS2); men are shown on the left, females on the right. The black and red closed circles represent DHS2 participants with telomere lengths greater and less than the 5th percentile, respectively. The mean MCV for the entire population is 88.9 fL; the mean MCV for the population with telomere lengths below the 5th percentile is 91.4 fL.

Shorter telomere lengths are associated with decreased RBC counts, increased MCV, increased hemoglobin content and increased RDW. These red cell parameters are tightly correlated with each other; the relationships between MCV and hemoglobin or RDW are especially significant for females (**Figure 2**). MCV is directly related to hemoglobin content and inversely related to RDW and to RBC count. Subjects with shorter telomere lengths tend to have RBCs of larger size; subjects with telomere lengths less than the 5th percentile have a mean MCV of 91.4 fL, whereas the mean MCV for the entire population is 88.9 fL. The MCV, hemoglobin, RDW and RBC counts for subjects with telomere lengths less than the 5th percentile are shown as red symbols in **Figure 2**. RBC count and hemoglobin are highly correlated with the inter-individual variability of MCV (**Table S4**). However, even after accounting for these closely inter-related parameters, telomere lengths remain significantly associated with MCV. After excluding RBC count and hemoglobin, we find significant associations between MCV and several demographic and clinical covariates using a multiple regression model (**Table 4**).

**Table 4:** Clinical Variables Associated with MCV Excluding RBC Count and Hemoglobin.

	Model adjusted for age, gender, and ethnicity(n = 3157, R^2^ = 0.0777)		Model adjusted for all covariates(n = 2643, R^2^ = 0.2225)	
	Beta (SE)	*P*-value	Beta (SE)	*P*-value
Age (per year)	0.0502 (0.0102)	8.7×10^−7^	0.0370 (0.0107)	0.0006
Gender				
Female	Reference	–	Reference	–
Male	1.6029 (0.2288)	3.0×10^−12^	0.1477 (0.2423)	0.5422
Ethnicity				
European Am. vs. African Am.	3.1648 (0.2496)	5.5×10^−36^	2.1917 (0.2660)	2.7×10^−16^
Hispanic vs. African Am.	1.6061 (0.3336)	1.5×10^−6^	1.0469 (0.3330)	0.0017
Hispanic vs. European Am.	−1.5588 (0.3553)	1.2×10^−5^	−1.1448 (0.3562)	0.0013
BMI			−0.1190 (0.0161)	1.7×10^−13^
Ln (Ave Relative T/S)			−2.4013 (0.4453)	7.6×10^−8^
Alcohol intake (g/day)			0.0474 (0.0099)	1.6×10^−6^
Smoking				
Never			Reference	–
Past smoking			0.7118 (0.2916)	0.0147
Current smoking			1.6046 (0.2962)	6.6×10^−8^
Iron			0.0518 (0.0032)	2.3×10^−55^

The effect sizes (betas) are reported as mean differences in MCV associated with a one-unit increase in a quantitative predictor, or with a particular category of a qualitative predictor compared to the reference group. Iron measurements were available for 2712 of the participants; the estimated coefficients from the full model are based on this subset.

Age, ethnicity, telomere length, BMI, current smoking, alcohol ingestion and iron explain over 22% of the variation in MCV. Iron is by far the most significant covariate (p = 2.3×10^−55^). Males have much higher iron levels. Addition of iron to the model attenuates the effect of gender. European American ethnicity is statistically associated with larger MCV, as compared with African Americans or Hispanics (p = 2.7×10^−16^ and 0.0013, respectively). European Americans tend to have the largest MCV, followed by HIspanic, then African American populations. Body mass index is a significant contributor to MCV. We find that subjects with larger BMI have smaller MCVs. For every 1-unit increase in BMI, there is a decrease of 0.12 fL in MCV. Smoking and alcohol intake increase red blood cell size [25], [26]; we also find that both are associated with MCV (p = 6.3×10^−8^ and 1.6×10^−6^, respectively). MCV is on average 1.60 fL larger in current versus never smokers. Alcohol intake of 14 g/day (approximately one drink per day) is associated with an increase of 0.65 fL of the MCV. Folate and vitamin B12 levels were not measured in the DHS2 population; however, total homocysteine levels were analyzed as a metabolic surrogate for folate and cobalamin deficiency [27]. We find that homocysteine levels do not add to the model as an independent predictor of MCV after correction for the covariates listed in **Table 4**. Telomere lengths are inversely related to MCV after accounting for all other covariates (p = 7.6×10^−8^). The effect of telomere length on MCV is comparable to the effect of alcohol intake or current smoking (partial R^2^ = 1.1% for telomere length, 0.9% for alcohol consumption and 1.1% for smoking). We find that the association between telomere length and MCV is seen for both subjects older and younger than the median age of 50 years, but is more robust for the older cohort (p = 9.2×10^−7^ for >50 years of age vs. p = 0.0006 for <50 years of age). We find no association between the *TERC* SNPs and RBC size. We find no evidence for interactions between telomere length, gender and ethnicity that significantly affect MCV.

## Discussion

Red blood cells transport oxygen necessary for cell respiration. They undergo a unique maturation process of discarding nuclei and organelles from reticulocytes and assuming a biconcave shape. During this process the red cells decrease in size. It was noted in 1972, during the early days of using automated Coulter machines to measure hematologic parameters, that mean MCV increases with age [28]. Macrocytosis is common; a RBC size of >100 fL is found in about 4% of the population [29]. It is often unexplained by low folate or vitamin B12 levels or abnormal thyroid and liver function tests [30]. It is more common in those older than 50 years of age and is associated with increased risk of myelodyspastic syndrome and worse outcomes after coronary interventions [29], [31], [32]. Differences of hematologic characteristics, including RBC size, have been studied in African Americans and European Americans and can only be partly explained by iron deficiency and the increased frequency of α-thalassemia deletion in African Americans [33]. The variability of MCV across populations, like other RBC characteristics, is not completely explained by genetic loci [34] or environmental exposures [25], [26], [35], [36].

The findings of this study suggest that MCV is inversely associated with telomere length and the magnitude of the effect of telomere lengths is comparable to the effect of alcohol intake or current smoking. Telomeres shorten in humans with each cell division due to incomplete lagging strand DNA synthesis and limited telomerase activity. Overall, telomere lengths are affected by starting set points, activity of telomerase in tissue progenitor cells, cell division and exposure to oxidative stress. The association between telomere length and the maximum proliferative potential of cord blood CD34+ hematopoietic progenitor cells is more robust for the erythroid lineage than the other myeloid lineages [37]. Perhaps this explains the significant associations between telomere length and multiple RBC parameters, but not total WBC count. Extremely short or critically short telomeres lead to senescence [38]. The very short telomere lengths found in patients with rare, inherited telomerase mutations lead to replicative senescence of progenitor cells and severe anemia. The not-so-short telomere lengths that are found in a population-based cohort may lead to milder degrees of replicative stress and less impaired maturation of the erythroid lineage. Members of this cohort with shorter telomere lengths have a mean RBC size that falls within the normal range and includes outliers with macrocytosis, as seen in **Figure 2**.

Different environmental stressors can affect red blood size. Drugs such as azathioprine and cyclophosphamide, which have cytotoxic bone marrow effects, can lead to an dose-dependent enlargement of RBCs [39], [40]. Replicative stress of erythroid progenitor cells from folate or cobalamin deficiency leads to nuclear and mitochondrial DNA instability and a reversible macrocytosis. Epidemiologic associations do not implicate a causative relationship. The cross-sectional observational design of this study does not allow us to determine whether short telomeres are related to MCV through a mechanism of reduced erythrocyte stem cell telomerase activity or as a marker of the effects of a lifetime of environmental damage to RBC progenitor cells. Regardless, the effect of telomere length on MCV increases with age.

Other studies have examined the relationship between telomere length and RBC parameters. The Asklepios study measured telomere lengths of ∼2500 healthy subjects aged 35–55 from two Belgian communities [41]. They found, like this study, that shorter telomere length were associated with lower RBC counts, larger RBC size and higher hemoglobin concentrations especially in men. Iron, which was found to be a significant covariate for MCV in this study, was not measured and the lack of this variable may have obscured the association in women. Two other studies have failed to find an association between telomere length and multiple hematological parameters. Both of these studies differ from the current one in that they evaluated only older subjects; one study evaluated subjects with a mean age of 71 years [42] and the other studied individuals >85 years [43]. The fuller range of telomere lengths and hematopoietic phenotypes measured across a population that spans seven decades distinguishes the current study.

Like the Asklepios study [41], we also find that obesity is negatively associated with RBC size but the basis for this association is not readily apparent. Higher BMI has been associated with nutritional deficiencies [44], but we find that this association holds after correcting for iron levels. Obesity has also been strongly associated with erythrocyte viscosity and alterations of erythrocyte membrane lipid fluidity [45], [46]. Genetic alterations of plasma lipoprotein levels in mice can lead to a failure of RBC maturation and lead to RBC macrocytosis [47]. While we did not directly measure erythrocyte viscosity, we find no association between MCV and cholesterol or triglycerides. Obesity can also lead to hypoventilation and increased production of RBCs through increased production of erythropoietin, which also was not directly measured in this study.

There are a number of differences between this study and other population-based studies of telomere length. Some of these differences may be due to ethnic diversity of this cohort; 51% of the participants are African American, 32% European American and 14% Hispanic. We find that telomere length declines on average by ∼10 basepairs per year. This estimated rate of attrition is less than other estimates, including both cross-sectional and longitudinal studies, that leukocyte telomeres shorten by ∼15–45 basepairs per year [18], [48], [49], [50], [51]. This may be related to the method of telomere length determination. Here we have used a quantitative PCR (qPCR) method for estimation of length of telomeres relative to a single copy gene that requires only a small amount of input genomic DNA (20 ng per reaction). It is a limitation of the PCR-based nature of this assay that measurements of telomere size may not be completely accurate, which would affect our estimate of telomere attrition in basepairs. Using a group of 466 subjects for which telomere length has been analyzed both by this assay and a Southern blot method of measuring telomere and subtelomeric lengths, we have converted the qPCR measured telomere lengths into kilobases. We and others have found that the measurement of telomere length by qPCR has a higher inter-assay coefficient of variation than the Southern blot method and correlates less reliably for individuals with longer telomere lengths [15], [52]. The observed mean CV for the qPCR measurement is lower for this study than the CVs reported for many others [16], [52], [53], [54].

We find that males have significantly shorter telomere lengths than females, as has been reported in many other studies. However, not all of our results are in agreement with other population-based telomere length association studies. First, we do not find a statistically significant difference between telomere lengths of African American and European American subjects. Other studies have reported no difference between these two ethnicities [55] or found differences in telomere lengths but in opposite directions [16], [54]. We do find that Hispanics have longer telomere lengths in comparison with African or European Americans, and this relationship remains significant after correction for all covariates. We find that current (but not former) smoking is associated with shorter telomere lengths after adjusting for age, gender and ethnicity. Others have reported a correlation between longer telomere lengths and amount of education and income [16], [56]; we also find a significant association between longer telomere length and years of education and income (shown in **Table S1**). In contrast to other studies [53], [57], [58], we do not find any association between telomere length and clinical measures of adiposity, such as BMI, waist circumference or measures of liver fat for women or men (data not shown). Two common variants located near the *TERC* gene have been found to be associated with short telomere lengths in British, European and European American cohorts [23], [24]. Here we find that both variants are associated with telomere length in the same direction as described in previous reports. However, we see this association for Hispanic Americans (both SNPs) and African Americans (rs2293607 only), with no statistically significant association in European Americans. It is possible that many of these differences are due to cohort size, differences in allele frequencies or population substructure.

There are several limitations to this study. Measures that are known to influence MCV such as reticulocyte count, thyroid stimulating hormone, folate, vitamin B12 and erythropoietin levels were not directly measured and so we are unable to directly adjust for these variables. Elevated homocysteine levels are a highly sensitive test of folate or cobalamin deficiency and we find that homocysteine levels are not associated with the MCV is this cohort after adjustment of other covariates. Factors that are associated with relatively small changes in telomere length variation may not be detected. Others have found that measurement of telomere length is highly dynamic during a person's lifetime [18], [59], so a cross-sectional study may not fully capture endogenous intra-individual variation. Some of the important predictors, such as smoking and alcohol consumption, were obtained from a questionnaire and could suffer from measurement error or reporting bias.

Heterozygous germline inactivating mutations in the genes encoding telomerase have been found in patients with a range of hematopoietic dysfunction, ranging from no or mild abnormalities to extreme pancytopenia [60]. Patients with dyskeratosis congenita represent an extreme phenotype with macrocytosis [61] and very short telomere lengths. The hematologic phenotype of these patients replicates the same trend found in this population-based study. Here we show that RBC size is inversely associated with telomere length, especially in older members of a large, multi-ethnic population. Further studies are needed to explore the underlying biological mechanism of macrocytosis of aging and to confirm the impact of these findings in the general population.

## Materials and Methods

### Study Population

This study was approved by the University of Texas Southwestern Medical Center Institutional Review Board. Written consent was obtained from all subjects. The Dallas Heart Study (DHS) is a population-based, probability sample of Dallas County residents, designed to include about 50% African American and 50% non-African American subjects. The study was initiated in 2000, and was transformed from a cross-sectional to a longitudinal study in 2007, when all participants who had submitted blood samples in the first study were invited for a repeat evaluation. The DHS2 cohort was augmented by voluntary participation from unrelated family members. It includes 2485 participants from the initial DHS study and 916 unrelated subjects to augment the size of the cohort (51% African American, 32% European American, 14% Hispanic, and 3% Other). Each participant completed a detailed survey, including questions about family income, level of formal education, alcohol consumption, smoking history and medical history with a focus on cardiovascular disease. Race or ethnic identity was self-reported according to a list of categories used in the US census. Complete blood count (CBC) analysis and measurement of telomere lengths were performed for a subset of DHS2 participants, 18–85 years of age, who submitted blood samples for genetic analysis (n = 3302). CBCs were performed using a Coulter LH750 hematology analyzer with direct measurement of the hemoglobin and RBC count. The MCV and the red blood cell distribution width (RDW) were derived from the RBC histogram. The study population for the present analysis includes 3157 African American, European American and Hispanic subjects with both CBC and telomere length measurements. Iron and homocysteine measurements are available for 2712 and 2030 participants, respectively.

### Telomere Length Analysis

Genomic DNA was isolated from circulating leukocytes with an Autopure LS (Qiagen, Valencia, CA). Quantitative PCR determination of telomere lengths was performed as previously described [15] with the following changes. Genomic DNA was stored at 25 ng/µl in DNA hydration solution (Qiagen) prior to its dilution to 2 ng/µl in water; 20 ng was added to the final 25 µl reaction. The final concentration of MgCl_2_ was 4 mM. Each sample was assayed in triplicate. The ratio of the copy number of telomere DNA to single-copy gene (T/S) was calculated using the formula T/S = 2^−ΔCt^ where ΔCt = Ct^telomere^ - Ct^albumin^, where Ct is the PCR threshold cycle. The relative T/S represents the ratio of the T/S for the experimental sample to the T/S for MCF7, a cultured cell line with very short telomeres. The average of at least two independent measurements is reported as the mean relative T/S; if its coefficient of variation (CV) was greater than 14%, the telomere length was measured again until the CV was ≤14% across all measurements. The observed mean CV for the mean relative T/S is 4.09% for 3302 samples.

Telomere lengths of 466 additional samples were measured using the above protocol; we had previously measured telomere lengths of these samples using a Southern blot method (Terminal Restriction Fragment Length, TRFL) [15]. There was a strong linear relationship between the logarithm of the mean relative T/S and TRFL measurements (r = 0.85, p<0.0001), as estimated by the linear regression equation:
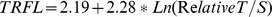
Thus, a log relative T/S ratio of 1 corresponds to a TRFL measurement of 4.5 Kb.

### Genotyping

Genotypes for two SNPs located in the 3' flanking region of the *TERC* gene: rs2293607 (+313 A>G) and rs12696304 (+1377 C>G) were determined using TaqMan® Pre-developed Assays for Allelic Discrimination (Applied Biosystems). The minor allele for rs12696304 in European-Americans was called a “G” to be consistent with dbSNP. A total of 3275 (out of 3317) individuals from the DHS2 were successfully genotyped for rs2293607 (call rate 98.7%), and these included 3110 participants of African American, European American or Hispanic ethnicities for whom telomere length and CBC analyses were performed. A total of 3263 (out of 3317) individuals were successfully genotyped for rs12696304 (call rate 98.4%), including 3100 with telomere length and CBC data.

### Statistical Methods

We used linear regression to estimate the relationship between relative T/S ratio and TRFL measurement of telomere length. Relative T/S ratios were log transformed prior to analysis to achieve normal distribution and constant variance. TRFL measurements were approximately normally distributed and were not transformed. Demographic and clinical variables across tertiles of telomere length were compared using chi-square tests for trend and logistic regression for categorical variables, and Jonckheere-Terpstra rank test for trend and linear regression for continuous variables (variables with a skewed distribution were first log transformed). Behavioral factors included in the study (smoking and alcohol ingestion) were analyzed as both categorical variables (current, former, never) and as continuous measures (pack-years smoked or grams/day of alcohol consumed). Linear regression was used to assess the relationship between clinical and demographic factors and MCV and to test the relationship between genetic variants and telomere length. Two-sided *p*-values <0.05 were considered statistically significant. All analyses were performed with the R software package, version 2.12.2 (www.r-project.org).

## Supporting Information

Figure S1 **Mean values of telomere length of DHS2 participants stratified by ethnicity and genotype.** (**A**) *TERC* SNP rs2293607 and (**B**) rs12696304. Telomere lengths are expressed as a natural logarithm of the ratio of the copy number of telomere DNA to a single-copy gene (Ln Relative T/S ratio). P-values were determined using a linear regression model with adjustment for age, gender and ethnicity. For rs2293607, the variant allele (G) is common in Hispanics (41%) and European Americans (25%), but less common in African Americans (7%). For rs12696304, the variant allele (G) is the minor allele in European Americans (28%) and the common allele in Hispanics and African Americans (53% and 56%, respectively). The rs2293607 (G) allele is associated with a modest decrease in telomere length in Hispanics and African Americans but not in European Americans; in contrast, the rs12696304 (G) allele is associated with shorter telomere lengths in Hispanics only.
(TIF)

Table S1 **Demographic and Clinical Characteristics of the DHS2 Stratified by Leukocyte Telomere Length Tertile.**
(DOCX)

Table S2 **Clinical characteristics of the Dallas Heart Study 2 participants stratified by TERC rs2293607 genotype and ethnicity.**
(DOCX)

Table S3 **Clinical characteristics of the Dallas Heart Study 2 participants stratified by TERC rs12696304 genotype and ethnicity.**
(DOCX)

Table S4 **Clinical Variables Associated with MCV.**
(DOCX)
