# Supplementary figures and images for: Red Blood Cell Size Is Inversely Associated with Leukocyte Telomere Length in a Large Multi-Ethnic Population
Source: PLoS One. 2012 Dec 4;7(12):e51046. doi: 10.1371/journal.pone.0051046 (PMC3514234; doi:10.1371/journal.pone.0051046)

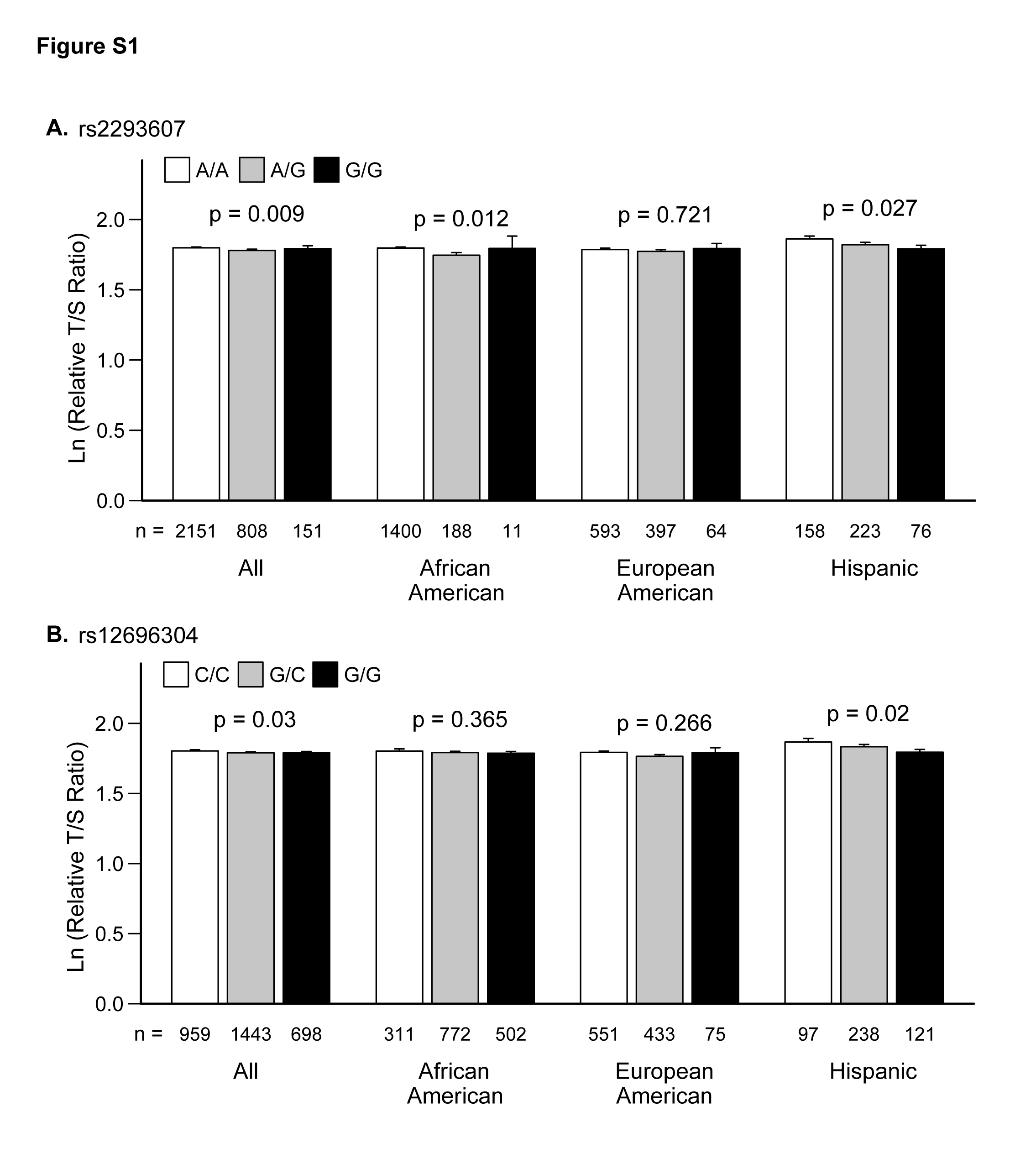

Supplement: Figure S1 — Mean values of telomere length of DHS2 participants stratified by ethnicity and genotype. (A) TERC SNP rs2293607 and (B) rs12696304. Telomere lengths are expressed as a natural logarithm of the ratio of the copy number of telomere DNA to a single-copy gene (Ln Relative T/S ratio). P-values were determined using a linear regression model with adjustment for age, gender and ethnicity. For rs2293607, the variant allele (G) is common in Hispanics (41%) and European Americans (25%), but less common in African Americans (7%). For rs12696304, the variant allele (G) is the minor allele in European Americans (28%) and the common allele in Hispanics and African Americans (53% and 56%, respectively). The rs2293607 (G) allele is associated with a modest decrease in telomere length in Hispanics and African Americans but not in European Americans; in contrast, the rs12696304 (G) allele is associated with shorter telomere lengths in Hispanics only. (TIF) [file pone.0051046.s001.tif]
