# Supplementary material for: Red Blood Cell Size Is Inversely Associated with Leukocyte Telomere Length in a Large Multi-Ethnic Population
Source: PLoS One. 2012 Dec 4;7(12):e51046. doi: 10.1371/journal.pone.0051046 (PMC3514234; doi:10.1371/journal.pone.0051046)
Supplement: Table S1 — Demographic and Clinical Characteristics of the DHS2 Stratified by Leukocyte Telomere Length Tertile. (DOCX) [file pone.0051046.s002.docx]

**Table S1. Demographic and Clinical Characteristics of the DHS2 Stratified by Leukocyte Telomere Length Tertile.**

|  | Short  (n = 1053) | Middle  (n = 1052) | Long  (n = 1052) | *P*-value | *P*-value adjusted for age, gender, and ethnicity |
| --- | --- | --- | --- | --- | --- |
| Telomere length (kb) | 4.14 – 6.02 | 6.02 – 6.53 | 6.53 – 9.20 |  |  |
| Age (years) | 52.7 ± 11.2 | 49.2 ± 10.9 | 48.3 ± 10.8 | <0.0001 | - |
| White Blood Subsets |  |  |  |  |  |
| Neutrophils (%) | 57.3 ± 10.2 | 57.9 ± 9.6 | 57.3 ± 9.7 | 0.8895 | 0.8769 |
| Lymphocytes (%) | 32.8 ± 9.3 | 32.2 ± 8.8 | 33.1 ± 8.9 | 0.3730 | 0.8798 |
| Monocytes (%) | 6.88 ± 2.21 | 6.75 ± 2.10 | 6.53 ± 2.03 | 0.0019 | 0.0293 |
| Eosinophils (%) | 2.58 ± 1.89 | 2.65 ± 2.20 | 2.60 ± 2.01 | 0.9666 | 0.2113 |
| Basophils (%) | 0.47 ± 0.23 | 0.48 ± 0.24 | 0.47 ± 0.21 | 0.3848 | 0.5209 |
| Income (%)  < $20,000  $20,000-$39,999  ≥ $40,000 | 29.1  28.9  41.9 | 24.3  29.3  46.5 | 26.1  26.9  47.0 | 0.1078  0.2669  0.0773 | 0.2111  0.0678  0.0042 |
| Highest Education (%)  Less than HS  Completed HS  College/Graduate School | 17.7  27.3  55.0 | 13.4  26.6  59.9 | 14.9  22.7  62.5 | 0.0791  0.0189  0.0004 | 0.0290  0.0072  <0.0001 |
| Education (years) | 13.3 ± 2.9 | 13.5 ± 2.8 | 13.6 ± 2.9 | 0.0056 | 0.0004 |
| Statins (%) | 19.6 | 17.3 | 16.3 | 0.0536 | 0.0804 |
| AST (U/L) | 24.3 ± 18.2 | 23.3 ± 12.9 | 23.7 ± 13.6 | 0.8633 | 0.1915 |
| ALT (U/L) | 22.6 ± 16.9 | 23.1 ± 17.8 | 23.3 ± 18.3 | 0.2751 | 0.0084 |
| ALP (U/L) | 77.0 ± 31.7 | 75.1 ± 28.4 | 72.1 ± 21.9 | 0.0002 | 0.0021 |
| Total bilirubin (mg/dL) | 0.59 ± 0.29 | 0.57 ± 0.29 | 0.56 ± 0.29 | 0.0074 | 0.0116 |
| Quantitative variables are shown as mean ± SD or median (1^st^ - 3^rd^ quartile). *P*-values are based on the Jonckheere-Terpstra test for trend or linear regression for continuous variables, and chi-square tests for trend or logistic regression for qualitative variables. Abbreviations: BMI, body mass index; HS, high school; AST, aspartate aminotransferase; ALT, alanine aminotransferase; ALP, alkaline phosphatase. | | | | | |
