# Supplementary material for: Red Blood Cell Size Is Inversely Associated with Leukocyte Telomere Length in a Large Multi-Ethnic Population
Source: PLoS One. 2012 Dec 4;7(12):e51046. doi: 10.1371/journal.pone.0051046 (PMC3514234; doi:10.1371/journal.pone.0051046)
Supplement: Table S2 — Clinical characteristics of the Dallas Heart Study 2 participants stratified by TERC rs2293607 genotype and ethnicity. (DOCX) [file pone.0051046.s003.docx]

**Table S2. Clinical characteristics of the Dallas Heart Study 2 participants stratified by TERC rs2293607 genotype and ethnicity**

|  | African American | | | | European American | | | | Hispanic | | | |
| --- | --- | --- | --- | --- | --- | --- | --- | --- | --- | --- | --- | --- |
|  | A/A | A/G | G/G | P-value | A/A | A/G | G/G | P-value | A/A | A/G | G/G | P-value |
| N | 1400 | 188 | 11 | 0.1004^†^ | 593 | 397 | 64 | 0.8693^†^ | 158 | 223 | 76 | 0.9230^†^ |
| Male, n (%) | 520 (37) | 60 (32) | 5 (45) | 0.3214 | 266 (45) | 190 (48) | 30 (47) | 0.4219 | 63 (40) | 96 (43) | 31 (41) | 0.7744 |
| Age (years) | 49.8±11.3 | 49.3±11.9 | 49.5±10.4 | 0.566 | 52.2±10.7 | 51.5±10.2 | 53.4±10.9 | 0.986 | 46.8±10.8 | 46.3±10.4 | 46.6±10.6 | 0.785 |
| BMI (kg/m^2^) | 32.5±8 | 32.3±7.8 | 30.4±4 | 0.5750 | 29.6±6.4 | 29.4±6.5 | 29.2±7 | 0.415 | 31.2±6.7 | 30.6±6.2 | 32.5±7.5 | 0.3220 |
| Income, n (%)  < $20,000  $20,000-$39,999  ≥ $40,000 | 431 (37)  356 (31)  377 (32) | 59 (38.1)  49 (32)  47 (30) | 0 (0)  3 (33)  6 (67) | 0.4417  0.835  0.5590 | 57 (11)  111 (21)  355 (68) | 33 (10)  69 (20)  240 (70) | 6 (10)  13 (22)  40 (68) | 0.6699  0.9622  0.7455 | 33 (26)  44 (35)  50 (39) | 48 (25)  77 (40)  66 (35) | 23 (35)  25 (38)  18 (27) | 0.1951  0.6015  0.0907 |
| Education (years) | 13.3±2.2 | 13.3±2.5 | 14.7±2.4 | 0.1917 | 14.8±2.5 | 14.7±2.4 | 14.7±2.3 | 0.4858 | 11.5±4 | 11.1±4.2 | 10.9±4.2 | 0.2981 |
| Smoking, n (%)  Never  Former  Current | 723 (53)  272 (20)  376 (27) | 110 (59)  29 (16)  46 (25) | 7 (64)  3 (27)  1 (9) | 0.091  0.4256  0.2296 | 306 (52)  170 (29)  107 (18) | 189 (49) 120 (31)  78 (20) | 32 (52)  13 (21)  17 (27) | 0.4415  0.646  0.124 | 89 (58)  36 (23)  29 (19) | 151 (69)  38 (17)  30 (14) | 48 (64)  15 (20)  12 (16) | 0.1297  0.3059  0.380 |
| Smoking (pack-years) ^†^ | 12 (6-24) | 13.5 (8-21) | 7.95 (-) | 0.9502 | 23 (11-37) | 22 (13-35) | 22 (10-44) | 0.774 | 12 (6-19) | 3.4 (2-8) | 6 (2-9) | 0.0020 |
| Drinking, n (%)  Never  Former  Current | 127 (9)  370 (27)  868 (64) | 15 (8)  44 (24)  124 (68) | 1 (9)  0 (0)  10 (91) | 0.6179  0.103  0.0705 | 24 (4)  97 (17)  460 (79) | 12 (3)  49 (13)  329 (84) | 6 (10)  9 (15)  47 (76) | 0.3904  0.1513  0.380 | 28 (18)  27 (17)  101 (65) | 44 (20)  34 (16)  141 (64) | 13 (17)  14 (19)  48 (64) | 0.8707  0.916  0.8242 |
| Alcohol intake (g/day) | 0.1 (0-2.8) | 0.1 (0-2.1) | 1.1 (0.4-4.2) | 0.582 | 0.6 (0.1-5.6) | 1.1 (0.1-8.4) | 0.6 (0-8.4) | 0.071 | 0.3 (0-6.2) | 0.2 (0-2.1) | 0.1 (0-2.3) | 0.0481 |
| Telomere length (kb) | 6.29±0.6 | 6.17±0.59 | 6.28±0.66 | 0.0123 | 6.26±0.55 | 6.23±0.57 | 6.28±0.65 | 0.7208 | 6.44±0.58 | 6.34±0.57 | 6.28±0.5 | 0.0272 |
| WBC count (× 10^9^/L) | 6.4±2.17 | 6.12±1.98 | 6.84±1.9 | 0.2531 | 6.89±2.08 | 6.93±1.95 | 7.21±1.94 | 0.228 | 6.87±1.92 | 6.83±1.72 | 6.94±1.93 | 0.8331 |
| RBC count (× 10^9^/L) | 4.53±0.52 | 4.47±0.51 | 4.56±0.44 | 0.384 | 4.6±0.45 | 4.63±0.42 | 4.66±0.45 | 0.3463 | 4.65±0.47 | 4.67±0.47 | 4.64±0.42 | 0.8455 |
| Hemoglobin (g/dL) | 13.2±1.7 | 13.1±1.5 | 13.2±1.3 | 0.921 | 14.2±1.5 | 14.3±1.3 | 14.4±1.4 | 0.140 | 13.9±1.7 | 14.2±1.5 | 13.9±1.7 | 0.896 |
| MCV (fL) | 87.5±7.3 | 88.1±6 | 87±4.6 | 0.2846 | 90.7±5.1 | 91.4±5 | 91.1±6.1 | 0.0993 | 88.9±6.6 | 89.5±5.1 | 88.3±7.1 | 0.6858 |
| RDW (%) | 14.5±1.8 | 14.2±1.3 | 14.8±1.5 | 0.0856 | 13.6±1.2 | 13.5±0.9 | 13.7±1.2 | 0.9117 | 14±1.6 | 13.7±1 | 13.9±1.7 | 0.308 |
| Platelet count (× 10^9^/L) | 255±72 | 251±68 | 271±81 | 0.53 | 244±61 | 250±64 | 254±64 | 0.0554 | 249±64 | 248±72 | 246±67 | 0.7142 |
| Iron (μg/dL) | 80.5±35.7 | 87.7±34.3 | 76.3±25.5 | 0.0097 | 95.7±35.7 | 97.1±36.4 | 99.8±34.7 | 0.409 | 88±37.1 | 94.9±38.8 | 99.2±52.8 | 0.0712 |
| AST (U/L) | 22.6±13.5 | 23.7±20.1 | 25.5±6 | 0.2719 | 23.8±10.6 | 25.1±22.2 | 23.7±8 | 0.9009 | 26.2±16.3 | 25.4±15.3 | 27±14.8 | 0.7715 |
| ALT (U/L) | 20.4±15 | 20.9±15.5 | 22±8.2 | 0.1906 | 24.5±18.6 | 24.3±16.6 | 24.5±11 | 0.795 | 26.6±17.5 | 28.7±25 | 31.9±24.8 | 0.362 |
| ALP (U/L) | 75.8±29.5 | 74.9±23.4 | 62.8±18.1 | 0.2917 | 69.5±21.6 | 70±21.7 | 70.9±20.8 | 0.447 | 80.8±28.4 | 82.1±37.4 | 83.4±27.1 | 0.4789 |
| Total bilirubin (mg/dL) | 0.54±0.29 | 0.55±0.23 | 0.58±0.21 | 0.237 | 0.6±0.29 | 0.6±0.27 | 0.57±0.27 | 0.3551 | 0.63±0.35 | 0.61±0.31 | 0.65±0.31 | 0.869 |
| Quantitative data are reported as mean±SD or median (1^st^ quartile – 3^rd^ quartile). *P*-values were calculated using linear regression for quantitative characteristics, and logistic regression for categorical variables. All models were adjustment for age and gender, ^†^*P*-values for deviation from Hardy-Weinberg equilibrium were determined using chi-square tests. Abbreviations: BMI, body mass index; AST, aspartate aminotransferase; ALT, alanine aminotransferase; ALP, alkaline phosphatase. | | | | | | | | | | | | |
