# Supplementary material for: Red Blood Cell Size Is Inversely Associated with Leukocyte Telomere Length in a Large Multi-Ethnic Population
Source: PLoS One. 2012 Dec 4;7(12):e51046. doi: 10.1371/journal.pone.0051046 (PMC3514234; doi:10.1371/journal.pone.0051046)
Supplement: Table S3 — Clinical characteristics of the Dallas Heart Study 2 participants stratified by TERC rs12696304 genotype and ethnicity. (DOCX) [file pone.0051046.s004.docx]

**Table S3. Clinical characteristics of the Dallas Heart Study 2 participants stratified by TERC rs12696304 genotype and ethnicity**

|  | African American | | | | European American | | | | Hispanic | | | |
| --- | --- | --- | --- | --- | --- | --- | --- | --- | --- | --- | --- | --- |
|  | C/C | C/G | G/G | P-value | C/C | C/G | G/G | P-value | C/C | C/G | G/G | P-value |
| N | 311 | 772 | 502 | 0.6466^†^ | 551 | 433 | 75 | 0.4420^†^ | 97 | 238 | 121 | 0.3482^†^ |
| Male, n (%) | 113 (36) | 290 (38) | 177 (35) | 0.6634 | 247 (45) | 209 (48) | 33 (44) | 0.5668 | 35 (36) | 108 (45) | 46 (38) | 0.8846 |
| Age (years) | 49.9±11.2 | 49.7±11.3 | 49.5±11.4 | 0.631 | 52.2±10.5 | 51.8±10.4 | 52.7±11.5 | 0.849 | 47.7±10.8 | 46.4±10.4 | 45.9±10.7 | 0.208 |
| BMI (kg/m^2^) | 32.8±7.6 | 32.2±8.2 | 32.5±7.7 | 0.561 | 29.6±6.4 | 29.5±6.4 | 28.6±6.7 | 0.303 | 30.6±6.2 | 30.8±6.5 | 32.2±7.1 | 0.0706 |
| Income, n (%)  < $20,000  $20,000-$39,999  ≥ $40,000 | 98 (38)  69 (27)  90 (35) | 231 (36)  212 (33)  191 (30) | 153 (36)  127 (30)  145 (34) | 0.6717  0.645  0.9790 | 54 (11)  103 (21)  332 (68) | 37 (10)  75 (20)  260 (70) | 7 (10)  15 (22)  45 (67) | 0.7172  0.9656  0.8398 | 19 (25)  30 (40)  26 (35) | 47 (23)  76 (38)  78 (39) | 37 (35)  41 (38)  29 (27) | 0.0716  0.7183  0.199 |
| Education (years) | 13.5±2.2 | 13.3±2.2 | 13.2±2.2 | 0.2564 | 14.8±2.4 | 14.7±2.4 | 14.7±2.2 | 0.5633 | 11.1±4.5 | 11.2±4.1 | 11.1±4 | 0.9212 |
| Smoking, n (%)  Never  Former  Current | 155 (51)  63 (21)  86 (28) | 401 (53)  137 (18)  214 (28) | 276 (56)  101 (20)  119 (24) | 0.227  0.8262  0.1288 | 287 (53)  159 (29)  96 (18) | 207 (49)  131 (31)  85 (20) | 38 (53)  13 (18)  21 (29) | 0.4473  0.337  0.0403 | 57 (60)  20 (21)  18 (19) | 151 (65) 51 (22)  30 (13) | 79 (66)  19 (16)  22 (18) | 0.4048  0.3264  0.955 |
| Smoking (pack-years) ^†^ | 9.6 (5-21) | 13 (7-22) | 12 (6-25) | 0.6605 | 23 (10-38) | 22 (13-35) | 21 (11-44) | 0.67 | 8 (6-16) | 5 (2-9) | 7 (2-15) | 0.1003 |
| Drinking, n (%)  Never  Former  Current | 35 (11)  87 (29)  183 (60) | 67 (9)  200 (27)  487 (65) | 41 (8)  121 (25)  325 (67) | 0.1735  0.280  0.0662 | 23 (4)  92 (17)  425 (79) | 13 (3)  53 (12)  359 (84) | 6 (8)  10 (14)  56 (78) | 0.5823  0.0976  0.218 | 17 (18)  20 (21)  59 (61) | 47 (20)  37 (16)  149 (64) | 21 (18)  18 (15)  81 (68) | 0.9928  0.3181  0.4355 |
| Alcohol intake (g/day) | 0.1 (0-2.8) | 0.1 (0-2.8) | 0.1 (0-2.8) | 0.778 | 0.6 (0.1-5.6) | 1.1 (0.1-8.4) | 0.6 (0-7.7) | 0.0378 | 0.1 (0-5.6) | 0.2 (0-2.8) | 0.2 (0-3.2) | 0.966 |
| Telomere length (kb) | 6.3±0.62 | 6.28±0.59 | 6.27±0.61 | 0.365 | 6.28±0.54 | 6.22±0.58 | 6.27±0.69 | 0.266 | 6.45±0.57 | 6.37±0.59 | 6.28±0.51 | 0.0196 |
| WBC count (× 10^9^/L) | 6.46±2.35 | 6.33±1.99 | 6.37±2.22 | 0.6763 | 6.84±1.97 | 6.99±2.11 | 7.04±1.95 | 0.199 | 7.08±2 | 6.73±1.7 | 6.92±1.88 | 0.5508 |
| RBC count (× 10^9^/L) | 4.53±0.51 | 4.51±0.51 | 4.54±0.54 | 0.526 | 4.6±0.45 | 4.63±0.42 | 4.61±0.45 | 0.6263 | 4.64±0.46 | 4.68±0.48 | 4.65±0.44 | 0.9153 |
| Hemoglobin (g/dL) | 13.2±1.7 | 13.1±1.7 | 13.2±1.6 | 0.329 | 14.2±1.5 | 14.3±1.3 | 14.2±1.4 | 0.318 | 13.9±1.7 | 14.2±1.6 | 13.9±1.6 | 0.510 |
| MCV (fL) | 87.3±6.9 | 87.6±7.5 | 87.7±6.6 | 0.4267 | 90.7±5 | 91.3±5 | 91.3±6.2 | 0.0666 | 88.9±6.8 | 89.4±5.3 | 88.5±6.7 | 0.5909 |
| RDW (%) | 14.4±1.8 | 14.5±1.8 | 14.5±1.6 | 0.691 | 13.6±1.2 | 13.5±1 | 13.7±1.2 | 0.8711 | 14±1.8 | 13.8±1.1 | 13.9±1.6 | 0.570 |
| Platelet count (× 10^9^/L) | 257±70 | 255±71 | 252±72 | 0.147 | 245±61 | 248±63 | 255±64 | 0.135 | 251±63 | 247±73 | 249±65 | 0.6925 |
| Iron (μg/dL) | 79±34.7 | 81.5±36.3 | 82.4±35.2 | 0.1642 | 96.2±35.8 | 96.7±36.1 | 99±34.2 | 0.606 | 88.8±39 | 92.8±38.1 | 97.9±47.8 | 0.110 |
| AST (U/L) | 23.3±12.1 | 22.6±16.1 | 22.7±12.9 | 0.378 | 23.7±10.6 | 25.4±21.8 | 23.7±7.7 | 0.5892 | 26.4±18.1 | 25.4±15.3 | 26.8±13.7 | 0.3327 |
| ALT (U/L) | 21.9±15.7 | 20±15.5 | 20.5±13.8 | 0.396 | 24.4±19 | 25.1±18.9 | 23.9±10.5 | 0.401 | 27.3±18.7 | 28±24.1 | 30.5±22.8 | 0.368 |
| ALP (U/L) | 75±23.3 | 75.9±33.8 | 75.6±22.9 | 0.6480 | 69.7±21.7 | 70.1±21.7 | 69.5±20.8 | 0.777 | 81.2±31.4 | 81.1±36.2 | 83.9±27 | 0.1845 |
| Total bilirubin (mg/dL) | 0.53±0.25 | 0.55±0.31 | 0.54±0.25 | 0.298 | 0.6±0.29 | 0.61±0.28 | 0.56±0.25 | 0.427 | 0.62±0.26 | 0.61±0.36 | 0.65±0.29 | 0.466 |
| Quantitative data are reported as mean±SD or median (1^st^ quartile – 3^rd^ quartile). *P*-values were calculated using linear regression for quantitative characteristics, and logistic regression for categorical variables. All models were adjustment for age and gender, ^†^*P*-values for deviation from Hardy-Weinberg equilibrium were determined using chi-square tests. Abbreviations: BMI, body mass index; AST, aspartate aminotransferase; ALT, alanine aminotransferase; ALP, alkaline phosphatase. | | | | | | | | | | | | |
