# Supplementary material for: Red Blood Cell Size Is Inversely Associated with Leukocyte Telomere Length in a Large Multi-Ethnic Population
Source: PLoS One. 2012 Dec 4;7(12):e51046. doi: 10.1371/journal.pone.0051046 (PMC3514234; doi:10.1371/journal.pone.0051046)
Supplement: Table S4 — Clinical Variables Associated with MCV. (DOCX) [file pone.0051046.s005.docx]

**Table S4. Clinical Variables Associated with MCV**

|  | Model adjusted for RBC count and Hemoglobin (R^2^ = 0.9142) | | Model adjusted for all covariates  (R^2^ = 0.9146) | | |
| --- | --- | --- | --- | --- | --- |
|  | Beta (SE) | *P*-value | Beta (SE) | *P*-value | |
| RBC Count | -16.2474 (0.1001) | 0 | -16.2082 (0.1004) | 0 |  |
| Hemoglobin | 5.2133 (0.0297) | 0 | 5.1992 (0.0298) | 0 |  |
| Ln (Ave Relative T/S) |  |  | -0.5086 (0.1325) | 0.00013 |  |
| The effect sizes (betas) are reported as mean change in MCV associated with a one-unit increase in a particular factor. | | | | | |
